# SLC10A3 drives glioblastoma progression by remodeling the immunosuppressive microenvironment and promoting M2 macrophage migration

**DOI:** 10.3389/fonc.2026.1742452

**Published:** 2026-05-13

**Authors:** Shuhui Chen, Daogang Qin, Hanyang Lin, Taohui Ding, Wei Liu, Ziqi Yan, Lingting Wang, Bo Kou

**Affiliations:** 1 Department of Otorhinolaryngology-Head and Neck Surgery, The First Affiliated Hospital of Xi’an Jiaotong University, Xi’an, Shaanxi, China; 2 Department of Comprehensive Radiation Oncology, Jiangxi Key Laboratory of oncology, Jiangxi Cancer Hospital, The Second Affiliated Hospital of Nanchang Medical College, Jiangxi Cancer Institute, Nanchang, Jiangxi, China; 3 Department of science and education, The First People’s Hospital of Jiangxia District, Hubei University of Medicine, Shiyan, Hubei, China; 4 Department of Thoracic Surgery, The Second Affiliated Hospital, Jiangxi Medical College, Nanchang University, Nanchang, China

**Keywords:** glioblastoma, immunosuppression, M2 macrophages, SLC10A3, therapeutic target, tumor microenvironment

## Abstract

**Introduction:**

Glioblastoma (GBM) is an aggressive brain tumor with poor prognosis, limited therapeutic options, and a highly immunosuppressive microenvironment. This study investigated the clinical significance and biological role of SLC10A3 in GBM progression and immune evasion.

**Methods:**

Bulk transcriptomic data from TCGA and CGGA cohorts were analyzed to assess SLC10A3 expression and prognostic value. Functional enrichment, single-cell RNA sequencing, and cell–cell communication analyses were performed to explore SLC10A3-related pathways and cellular distribution. In vitro and in vivo experiments were conducted to evaluate the effects of SLC10A3 knockdown on GBM malignant phenotypes and macrophage recruitment.

**Results:**

SLC10A3 was significantly overexpressed in GBM and associated with poor prognosis. Enrichment analyses linked SLC10A3 to PI3K-Akt, NF-κB, HIF-1 signaling, macrophage infiltration, and T-cell suppression. Single-cell analysis showed SLC10A3 enrichment in tumor-associated astrocytes and macrophages, with enhanced astrocyte–macrophage crosstalk through MIF, MDK, and extracellular matrix remodeling pathways. SLC10A3 knockdown inhibited GBM cell proliferation, migration, and invasion, induced cell cycle arrest and apoptosis, reduced M2 macrophage migration, and suppressed xenograft tumor growth.

**Discussion:**

SLC10A3 may promote GBM aggressiveness and immune evasion by regulating malignant phenotypes and macrophage-associated immunosuppression, suggesting its potential as a therapeutic target for GBM.

## 1 Introduction

Glioblastoma (GBM) is the most malignant type of glioma, usually originates from astrocytes. Its main characteristics include rapid growth, strong invasiveness, and high recurrence rate. Most patients still have poor prognosis despite undergoing surgery and chemotherapy (1–3). Although the standard treatment regimen (surgical resection combined with concurrent chemoradiotherapy with temozolomide) has been used for more than a decade, the high heterogeneity of tumors leads to prominent problems of chemoresistance, and the median survival time of patients is still less than 15 months (4). The tumor microenvironment plays a crucial role in this disease. Current immunotherapeutic approaches targeting the immune microenvironment have made significant progress in solid tumors such as lung cancer and melanoma (5). However, in GBM, owing to the presence of an immunosuppressive microenvironment, immunotherapy only elicits a response in a small number of GBM patients (6). This current situation indicates that key regulatory factors in the GBM immune microenvironment are of great significance for the development of novel treatment strategies.

SLC10A3 is a member of the SLC10 family, which is associated with the human X chromosome and is a housekeeping protein (7), its specific function remains unclear. Previous studies have shown that it plays an important role in cancer progression. A previous study by Chen et al. using massively parallel signature sequencing (MPSS) and sequencing by synthesis (SBS) demonstrated that SLC10A3 is related to the chemosensitivity of ovarian cancer patients (8). In addition, research by Mabel Seto indicated that SLC10A3 is associated with the development of the nervous system and neuroinflammation (9). Moreover, Bangting Wang et al. showed that SLC10A3 is highly expressed in colorectal cancer and is associated with poor prognosis (10). Recently, a study by Yang He showed that it is highly expressed in low-grade gliomas, and its high expression is associated with a poor prognosis. Also, in the tumor microenvironment, the expression of SLC10A3 is related to the expression of macrophages and T cells (11). Through large-scale single-cell sequencing, the types of cancer cells in glioblastoma have been identified, among which astrocytes are the predominant type of cancer cells (12). Analysis of the single-cell sequencing data in the Gene Expression Omnibus (GEO) database revealed that SLC10A3 was significantly highly expressed in astrocytes and macrophages, and the cell communication between astrocytes with high SLC10A3 expression and macrophages was significantly enhanced. However, the specific roles of SLC10A3 in GBM progression and in the immune microenvironment remain unclear and require further investigation.

In this study, we integrated bulk transcriptome data from The Cancer Genome Atlas (TCGA) and the Chinese Glioma Genome Atlas (CGGA), and combined it with single-cell sequencing data analysis to systematically analyze the expression pattern and clinical significance of SLC10A3 in GBM for the first time. In addition, through an *in vitro* co-culture experiment of GBM cells and macrophages, it was determined that GBM cells with high SLC10A3 expression could significantly enhance the migration ability of M2 macrophages, suggesting that SLC10A3 may drive tumor progression by remodeling the immunosuppressive microenvironment. In conclusion, these results not only suggest that SLC10A3 may serve as a potential therapeutic target, although further studies are needed to validate its translational relevance but also provide a theoretical basis for the development of combination therapies targeting the interaction between tumors and immune cells.

## 2 Materials and method

### 2.1 Bulk transcriptome and single-cell data collection

Glioblastoma (GBM) samples were obtained from The Cancer Genome Atlas (TCGA) and the Chinese Glioma Genome Atlas (CGGA) databases (13). The GBM dataset was downloaded from TCGA, and 152 samples were retained after excluding those lacking clinical information on survival time and status. The CGGA693 dataset was acquired from CGGA and comprised 235 GBM samples. Additionally, pan-cancer RNA-seq data were downloaded from the UCSC XENA platform (https://xenabrowser.net/datapages/) and normal tissue RNA-seq data were obtained from the Genotype-Tissue Expression (GTEx) project. Single-cell RNA sequencing (scRNA-seq) data for 40 GBM samples were retrieved from the GEO database (GSE182109; https://www.ncbi.nlm.nih.gov/geo/) (14).

### 2.2 SLC10A3 expression and its prognostic relevance in glioma

Differential expression analysis of SLC10A3 between tumor and normal tissues was performed using RNA-seq data from the UCSC XENA and GTEx database. Comparative analysis of SLC10A3 expression in GBM and normal brain tissues was conducted using TCGA and GTEx datasets. Immunohistochemical (IHC) images of SLC10A3 in GBM and normal brain tissues were obtained from the Human Protein Atlas database (https://www.proteinatlas.org/) (15, 16). Survival information from the TCGA and CGGA cohorts was extracted to investigate the association between SLC10A3 mRNA expression and patient prognosis. Kaplan-Meier survival analysis was performed by stratifying the samples into high- and low-expression groups based on the median SLC10A3 mRNA expression level in TCGA. For CGGA data, the optimal survival cutoff was determined using the Survminer R package, followed by corresponding survival analysis.

### 2.3 Enrichment analysis

GBM samples from TCGA were divided into high- and low-expression groups based on the median SLC10A3 expression. Differential gene expression analysis was conducted using the limma package (|log2FC| > 0.8, P < 0.05), and 203 significantly differentially expressed genes were identified. Gene Set Enrichment Analysis (GSEA) was performed using the c2.cp.biocarta.v2023.1.Hs.entrez.gmt gene set from the MSIGDB database (https://www.gsea-msigdb.org/gsea/index.jsp). Gene Ontology (GO) enrichment analysis, including biological processes (BP), molecular functions (MF), and cellular components (CC), as well as Kyoto Encyclopedia of Genes and Genomes (KEGG) pathway analysis, were implemented using the clusterProfiler R package (17, 18).

### 2.4 Single-cell sequencing analysis

The 40 GBM scRNA-seq samples were processed using Python package Scanpy (19). Doublet cells were removed using the Scrublet method and further filtered based on surface marker genes (e.g., exclusion of CD68+/CD3E+ co-expressing cells) (20). The quality control criteria were as follows. Removal of cells expressing fewer than 200 genes. Exclusion of genes detected in fewer than 3 cells. Retention of cells with gene counts <5,000. Retention of cells with mitochondrial gene percentages <5%.Data integration was performed using scVI, a deep learning-based method (21). Post-integration, the “Scanpy.pp.neighbors” function was applied to construct a k-nearest neighbor graph, and dimensionality reduction was visualized using UMAP (Scanpy.tl.umap). The cell types were annotated using canonical marker genes. In this study, cell type annotation of single-cell transcriptome data was performed based on classical specific gene markers of various cell types in the glioma microenvironment. The specific markers are as follows: astrocytes (GFAP, AQP4, SLC1A2), oligodendrocytes (MOBP, MBP, PLP1, MAG), oligodendrocyte precursor cells (OPCs, PDGFRA, VCAN, CSPG4), macrophages (CD86, CD68, CD163), microglia (P2RY12, TMEM119, CX3CR1), T cells (CD3D, CD3E, CD3G), natural killer (NK) cells (NKG7, GNLY, KLRF1), B cells (MS4A1, CD79A, CD19), endothelial cells (CD34, CLDN5, CDH5), and mural cells (CD248, FOXF2, RGS5).

### 2.5 Immune infiltration analysis

The tumor immune microenvironment of high-grade gliomas in TCGA was evaluated using seven algorithms (CIBERSORT (22), EPIC (23), ESTIMATE (24), MCP-counter (25), QuantiSEQ (26), TIMER (27), xCELL (28)) implemented in the IOBR R package (29), with results visualized via heatmaps. Single-sample GSEA (ssGSEA) from the GSVA package (30) quantified enrichment scores for 24 immune cell types (gene sets from TISIDB: http://cis.hku.hk/TISIDB/). Spearman correlation analysis was performed to assess the association between SLC10A3 expression and immune scores. The differential expression of immune checkpoint inhibitors and immunostimulators between SLC10A3 expression groups was analyzed, and their correlations with SLC10A3 levels were quantified.

### 2.6 Mutation analysis

TCGA GBM mutation data were analyzed using the maftools R package (31). The top 20 mutated genes in the high- and low-SLC10A3 expression groups were visualized, and intergroup mutation differences were displayed using forest plots.

### 2.7 Clinical samples and study approval

For expression analysis, all fresh glioma samples and normal tissue specimens (from patients underwent brain tissue resection due to craniocerebral injury, and only tissues without obvious necrosis or inflammation were selected through pathological examination) were obtained from the Jiangxi Branch of the National Clinical Medical Research Center for Malignant Tumors (Jiangxi Cancer Hospital Biobank). After surgical resection, samples were washed with PBS and stored in liquid nitrogen for subsequent analysis. None of the patients had received preoperative chemotherapy or radiotherapy before the study. All experimental protocols were approved by the Ethics Committee of Jiangxi Cancer Hospital (Approval No. 2024ky051), and written informed consent was obtained from all enrolled patients.

### 2.8 Cell lines and cell culture

The glioma cell lines used in the experiments, U87MG, U251, T98G, U118, and HS683, were purchased from Shanghai FuHeng Biotechnology Co., Ltd. The cells were cultured in high-glucose DMEM (HyClone, USA), supplemented with 10% FBS (Sanofi TFB TFBC, Shenzhen) and 1% penicillin-streptomycin. The cultures were maintained in a humidified incubator at 37 °C and 5% CO2. All glioma cell lines (U87MG, U251, T98G, U118, HS683) were authenticated by short tandem repeat (STR) profiling within 6 months before the experiment, and the authentication results were consistent with the standard STR database; All cells used in this study were cultured for less than 20 passages; Mycoplasma contamination was tested negative by PCR before each experiment.

### 2.9 Generation and differentiation of M2 macrophages

THP-1 cells were treated with 100 ng/mL PMA for 24 hours (Med ChemExpress, USA) to induce M0 macrophage differentiation. The cells were then stimulated with IL-13 (20 ng/mL, R&D Systems) and IL-4 (20 ng/mL, R&D Systems) for 48 hours to promote their differentiation into M2 macrophages. Subsequently, cells were harvested and prepared as a single-cell suspension for flow cytometry. Prior to staining, cells were incubated with a species-matched anti-CD16/32 antibody (TruStain fcX, BioLegend) on ice for 10 min to block Fc receptors. Cells were then stained with APC-conjugated anti-CD206 and PE-conjugated anti-CD163 antibodies (1:100 dilution) for 30 min on ice in the dark. After staining, cells were washed with PBS containing 1% FBS and analyzed by flow cytometry.

### 2.10 Immunofluorescence

Cells were first seeded onto chamber slides, fixed with 4% paraformaldehyde and permeabilized with 0.1% Triton X-100. The samples were then blocked with 5% BSA at 25 °C for 1 hour. The cells were then incubated with the primary antibody overnight at 4 °C. After three washes with PBS, the slides were incubated with secondary antibody at 25 °C for 30 minutes. Finally, the nuclei were stained with DAPI for easy observation under a microscope.

### 2.11 Co-culture assay for M2 macrophage migration

Cell migration assays were performed using 6-well Transwell plates (Corning, USA). U87MG and U251 cells (5×10^5) from the si-NC and si-SLC10A3 groups were seeded in the lower chamber, while 5×10^5 M2 macrophages were added to the upper chamber. After co-culturing for 48 hours, M2 macrophages that migrated to the lower side of the membrane were fixed with 4% paraformaldehyde and stained with 0.1% crystal violet.

### 2.12 Cell transfection

Targeted small interfering RNA (siRNA) against SLC10A3 and siRNA-NC were purchased from RiboBio (Guangzhou, China). Glioma cells were seeded in 6-well plates at 40%-50% confluence. After 12 hours of cell attachment, transfection was performed using Lipofectamine^®^ RNAiMAX Transfection Reagent (Invitrogen, Carlsbad, CA, USA) at a final concentration of 100 nM, according to the manufacturer’s instructions. The growth medium was antibiotic-free. The siRNA sequences were as follows: siRNA-SLC10A3-#1: GGAGAGACTTCTGCATCAA; siRNA-SLC10A3-#2: GCGTGCTGATCAAGTCCAA.

### 2.13 RNA isolation and reverse transcription-PCR detection

Total RNA was extracted from glioma cells and tissues using TRIzol reagent (Takara, Dalian, China). The collected RNA was dissolved in 10 µl DEPC-treated water, and RNA concentration and purity were assessed by measuring the 260/280 nm ratio. Complementary DNA (cDNA) was synthesized using the PrimeScript™ RT Reagent Kit with gDNA Eraser (Takara, Dalian, China) according to the manufacturer’s instructions. Quantitative real-time polymerase chain reaction (qRT-PCR) was performed using TB Green^®^ Premix Ex Taq™ II (Takara, Dalian, China) on the Bio-Rad CFX96 Touch Real-Time PCR Detection System (Bio-Rad Laboratories Inc.). GAPDH was used as an endogenous control, and target gene expression levels were calculated by the 2^-ΔΔCT method. Each experiment was repeated thrice, and each sample was analyzed in two parallel wells. The primers for SLC10A3 were: 5′-TGATTGAGGAGCGGAGAGA-3′ and R: 5′-GCAGGTAGAGGATTGGGTTT-3′. The primers for GAPDH were: F: 5′-CCCATCACCATCTTCCAGGAG-3′ and R: 5′-GTTGTCATGGATGACCTTGGC-3′.

### 2.14 Western blot detection

Cells were transfected with siRNA to knock down the target genes. After 72 hours of transfection, the cells were harvested and lysed in RIPA buffer (Beyotime, Shanghai, China) containing 1% proteinase inhibitor cocktail (Beyotime, Shanghai, China). The lysates were centrifuged at 13,000×g for 15 minutes at 4 °C, and the supernatant was collected. The protein concentration was measured using a BCA protein assay kit (TIANGEN, Beijing, China). The supernatant was denatured with 6× loading buffer (TransGen Biotech, Beijing, China) and heated at 100 °C for 10 minutes. The samples were then separated by SDS-PAGE and transferred to a polyvinylidene difluoride (PVDF) membrane. The membrane was blocked for 2 hours with 5% non-fat milk and incubated overnight with primary antibodies at 4 °C with the following primary antibodies: SLC10A3 (Proteintech, 60004-1-Ig, Dilution ratio: 1:500); CD206 (AffinitY, DF4149, Dilution ratio: 1:500); CD163 (AffinitY, DF8235, Dilution ratio: 1:500); GAPDH (Proteintech, Dilution ratio: 1:200000) as an internal control. After washing, membranes were then incubated with species-matched secondary antibodies for 2 hours. The antibodies were purchased from CST Biotechnology (Boston, MA, USA). The bands were detected using an ECL detection kit (Shanghai YaMei) and visualized using a ChemiDoc Touch Imaging System (ChemiDoc XRS+). GAPDH was used as an internal control.

### 2.15 Cell proliferation and colony formation assays

The effects of gene manipulation were assessed using MTS and colony formation assays to explore the proliferative potential of GBM cells 48 hours after transfection with SLC10A3 siRNA. Briefly, the cells were seeded in 96-well plates at a density of 2000 cells per well. Optical density (OD) was measured every 24 hours for five consecutive days. MTS solution (CellTiter 96 AQueous One Solution Reagent, Promega, Madison, WI, USA) was mixed with serum-free medium at a 1:9 ratio, according to the manufacturer’s protocol. The mixture was then added to each well at a volume of 100 µl and incubated at 37 °C for 30 minutes. The OD was measured at 490 nm using a microplate reader (SpectraMAX ID3). For the colony formation assay, cells were seeded at a density of 2000 cells per well in 6-well plates and cultured in high-glucose DMEM supplemented with 10% FBS. Cells were allowed to grow for two weeks, and the medium was changed every three days. After 14 days of culture, cells were fixed with 4% paraformaldehyde and stained with 0.1% crystal violet. The colonies were imaged and counted. All experiments were performed in triplicate, with three replicates per group.

### 2.16 Flow cytometry analysis

Apoptotic cell populations were quantitatively analyzed using an Annexin V-FITC Apoptosis Detection Kit (Beyotime, Shanghai, China). After 48 hours of transfection, cells were collected and digested with trypsin without EDTA, followed by centrifugation to remove the supernatant. The cell pellet was washed with PBS centrifuged again, and the supernatant was discarded. The cells were then resuspended in annexin fluorescein isothiocyanate V-FITC binding buffer. According to the manufacturer’s instructions, cells were treated with Annexin V-FITC and PI staining solution and incubated for 10–15 minutes at room temperature in the dark. Finally, the cells were analyzed using an FC 500 flow cytometer (Beckman Coulter, Brea, California, USA).

### 2.17 Animal models

All animal experiments were conducted in accordance with the ethical guidelines provided by Nanchang Leyou Biotechnology Co., Ltd. (approval No. RYE2024031201; Animal Experiment License No. SYXK (Gan) 2023-0002). Female BALB/c nude mice (4–6 weeks old) were purchased from Hangzhou Ziyuan Laboratory Animal Technology Co., Ltd. (Zhejiang, China). For the *in vivo* xenograft model, mice were randomly divided into two groups (n=6). As described previously, stable U87MG cell lines (sh-NC and sh-SLC10A3) were dispersed to form a single-cell suspension and subcutaneously injected into each mouse at 5×10^6 cells. Tumor volumes were measured regularly, and after 3 weeks of tumor inoculation, the mice were euthanized (tumor volume should be <1500 mm^3). Tumor tissues were then harvested and divided into two parts: one portion was stored in liquid nitrogen, and the other was embedded in paraffin for subsequent immunohistochemistry experiments. Control lentivirus (sh-NC) and SLC10A3 lentivirus (LV-SLC10A3-shRNA) were purchased from Shanghai HanHeng Biotechnology Co., Ltd. (Shanghai, China).

### 2.18 Data analysis

All statistical analyses were performed using R (version 4.4.1) and Python (version 3.11). A P-value < 0.05 was considered statistically significant. The normality of continuous variables was assessed using the Shapiro-Wilk test and Q-Q plots. For intergroup comparisons, the Student’s t-test was applied for normally distributed variables, while the Wilcoxon rank-sum test was used for non-normally distributed variables. Statistical correlations between continuous variables were evaluated using Spearman’s correlation analysis. In cases of multiple comparisons, such as differential expression analysis of immune genes or multi-group comparisons in cell experiments, P-values were adjusted using either the Bonferroni correction or the false discovery rate (FDR) method. Survival probabilities were estimated using the Kaplan-Meier method, with group differences compared via the log-rank test. Additionally, Cox proportional hazards regression models were employed to calculate hazard ratios (HR) and their corresponding 95% confidence intervals (95% CI).

## 3 Results

### 3.1 Expression of SLC10A3 in pan-cancer and glioblastoma patients

**Figure 1:**
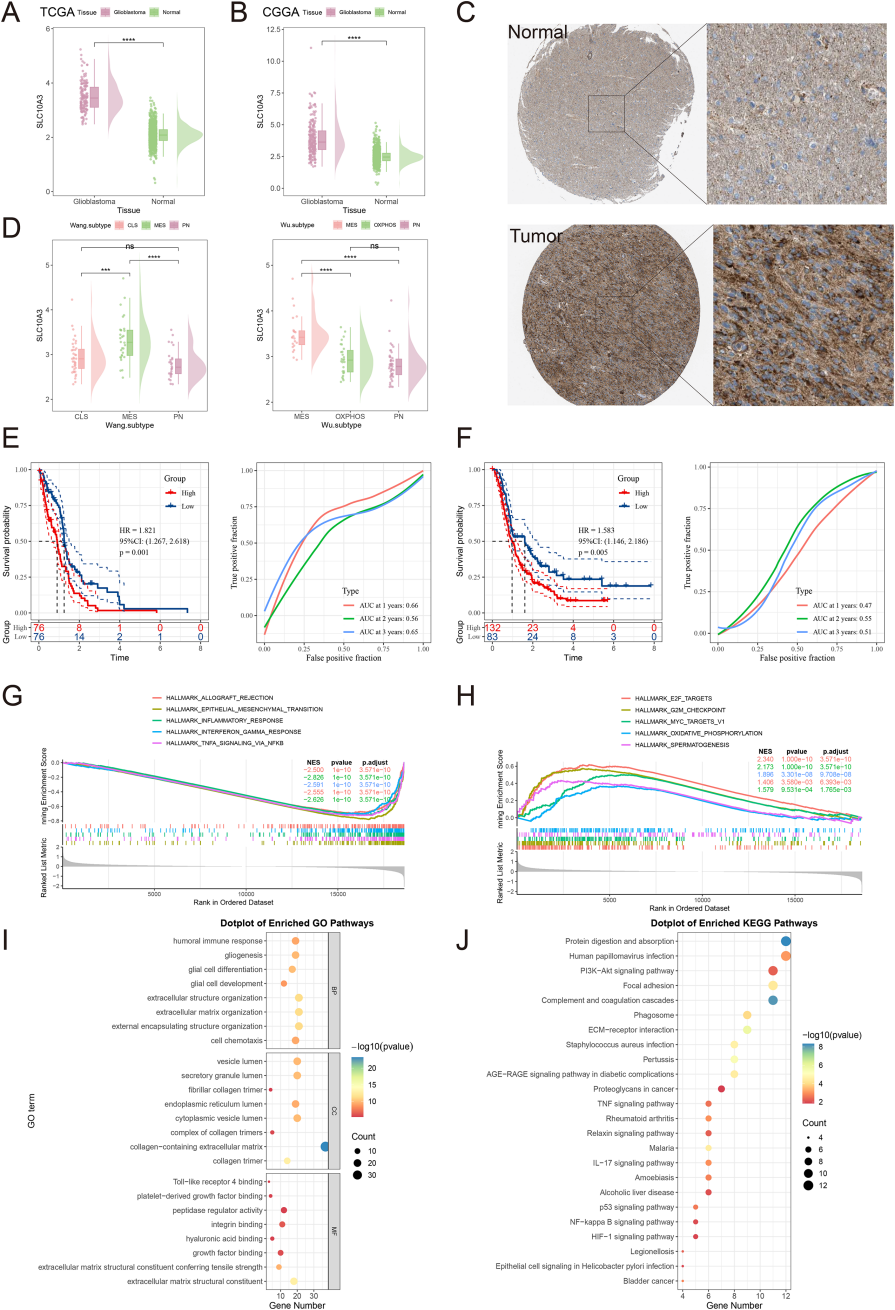
Differential expression levels of the SLC10A3 gene in glioblastoma (GBM) and enrichment analysis. **(A, B)** Expression of SLC10A3 in tumor tissue and normal tissue (TCGA and CGGA). **(C)** Representative immunehistochemistry (IHC) images of normal control group and tumor group. **(D)** SLC10A3 exhibits subtype-specific high expression in the mesenchymal (MES) subtype of glioblastoma (GBM). The expression pattern of SLC10A3 was analyzed across two independent TCGA GBM molecular classification systems: Wu et al. (subtypes: MES, proneural [PN], oxidative phosphorylation [OXPHOS]) and Wang et al. (subtypes: MES, PN, classical [CLS]). In both classification systems, SLC10A3 expression was significantly elevated in the MES subtype compared to the other subtypes. **(E)** Kaplan-Meier survival curves of overall survival in TCGA GBM cohort stratified by SLC10A3 expression and ROC curve. **(F)** Kaplan-Meier survival curves of overall survival in CGGA GBM cohort stratified by SLC10A3 expression and ROC curve. **(H, I)** GSEA pathway enrichment analysis. **(G)** GO enrichment analysis. **(K)** KEGG enrichment analysis. (*p < 0.05, **p < 0.01, ***p < 0.001, ****p < 0.0001).

Analysis of TCGA and GTEx data revealed significantly elevated SLC10A3 mRNA expression in 20 of 33 cancer types, including glioblastoma (GBM), low-grade glioma (LGG), adrenocortical carcinoma (ACC), breast cancer (BRCA), cervical squamous cell carcinoma (CESC), cholangiocarcinoma (CHOL), colon adenocarcinoma (COAD), diffuse large B-cell lymphoma (DLBC), esophageal carcinoma (ESCA), head and neck squamous cell carcinoma (HNSC), kidney renal clear cell carcinoma (KIRC), hepatocellular carcinoma (LIHC), lung squamous cell carcinoma (LUSC), pancreatic adenocarcinoma (PAAD), rectal adenocarcinoma (READ), stomach adenocarcinoma (STAD), testicular germ cell tumors (TGCT), and thymoma (THYM) (**Supplementary Figure 1A**). The overexpression of SLC10A3 in GBM was further validated using TCGA and CGGA databases (P < 0.05; **Figures 1A, B**). Immunohistochemical (IHC) analysis from the HPA database additionally demonstrated higher SLC10A3 protein levels in GBM tissues than in normal brain tissues (**Figure 1C**). We integrated the molecular classification systems of TCGA glioblastoma (GBM) from two independent studies, and in both systems (32, 33), we observed a significant subtype-specific expression pattern of the SLC10A3 gene. In the classification proposed by Min Wu et al., GBM was divided into mesenchymal (MES), proneural (PN), and oxidative phosphorylation (OXPHOS) subtypes, and the results showed that the expression level of SLC10A3 in the MES subtype was significantly higher than that in the PN and OXPHOS subtypes. In the classification system by Wang et al., GBM was categorized into three subtypes, namely MES, PN, and classical (CLS) subtypes, which further verified the tendency of SLC10A3 to exhibit subtype-specific high expression in the MES subtype(**Figure 1D**).

### 3.2 Prognostic significance of high SLC10A3 expression in glioblastoma and pan-cancer cohorts

Survival analysis of the TCGA (n=152) and CGGA (n=235) cohorts revealed that GBM patients with high SLC10A3 expression exhibited significantly shorter overall survival (TCGA: HR = 1.821, P = 0.001; CGGA: HR = 1.583, P = 0.005; **Figures 1E, F**). The 1-, 2-, and 3-year ROC curve AUC values for SLC10A3 in TCGA were 0.66, 0.56, and 0.65, respectively, while CGGA yielded AUCs of 0.47, 0.55, and 0.51 (**Figures 1E, F**). In addition, univariate and multivariate regression analyses of the TCGA GBM cohort data showed that SLC10A3 is an independent prognostic factor (HR = 1.67, 95%CI=1.135-2.457, P = 0.009)(**Supplementary Figure 1B**). These findings suggest that SLC10A3 is a potential prognostic biomarker for gliomas. Furthermore, SLC10A3 was identified as a high-risk prognostic marker in six additional cancer cohorts: adrenocortical carcinoma (ACC), pancreatic cancer (PAAD), renal cell carcinoma (KIRC), low-grade glioma (LGG), acute myeloid leukemia (LAML), and colon cancer (COAD) (**Supplementary Figures 1C–I**).

### 3.3 Functional enrichment of SLC10A3 in glioblastoma

To identify the functional networks of differentially expressed genes (DEGs) associated with high and low SLC10A3 expression in gliomas, we first performed Gene Set Enrichment Analysis (GSEA). The results demonstrated that the HALLMARK ALLOGRAFT REJECTION, HALLMARK EPITHELIAL MESENCHYMAL TRANSITION, HALLMARK INFLAMMATORY RESPONSE, HALLMARK INTERFERON GAMMA RESPONSE, and HALLMARK TNFA SIGNALING VIA NFKB signaling pathways were downregulated in the high-SLC10A3 expression group (**Figure 1H**). By contrast, the HALLMARK E2F TARGETS, HALLMARK G2M CHECKPOINT, HALLMARK MYC TARGETS V1, HALLMARK OXIDATIVE PHOSPHORYLATION, and HALLMARK SPERMATOGENESIS pathways were upregulated in the high-SLC10A3 group (**Figure 1I**). Furthermore, to investigate the biological functions of the most significantly altered genes in the SLC10A3 high- and low-expression groups, we conducted Gene Ontology (GO) and KEGG pathway enrichment analyses. GO-term analysis indicated that SLC10A3-associated genes were mainly involved in gliogenesis, glial differentiation, extracellular matrix (ECM) organization, and cell chemotaxis; these genes were also associated with molecular functions such as collagen trimer formation, integrin binding, and ECM structural components (**Figure 1G**). KEGG pathway analysis demonstrated that SLC10A3-related genes were predominantly enriched in PI3K-Akt signaling, phagosome formation, proteoglycans in cancer, TNF signaling, p53 pathway, NF-κB signaling, and HIF-1 pathway (**Figure 1K**). In addition, we also analyzed the correlation between SLC10A3 and genes related to epithelial-mesenchymal transition (EMT) and extracellular matrix (ECM) remodeling — such as SNAI1, SNAI2, TWIST1, VIM, FN1, COL1A1/COL3A1, LAMC1, TNC, MMP9, and ITGA5/ITGA2/ITGB1. We found that SLC is significantly positively correlated with these genes, which indicates that the mesenchymal transition and matrix reorganization programs are enhanced (**Supplementary Figure 2A**). Using the Ivy Glioblastoma Atlas Project (Ivy-GAP) dataset, we analyzed the regional expression patterns of SLC10A3. The results show that SLC10A3 is enriched in Hyperplastic blood vessels and Microvascular proliferation, consistent with features of ECM remodeling (**Supplementary Figure 2B**).

### 3.4 Single-cell molecular characterization of SLC10A3

**Figure 2:**
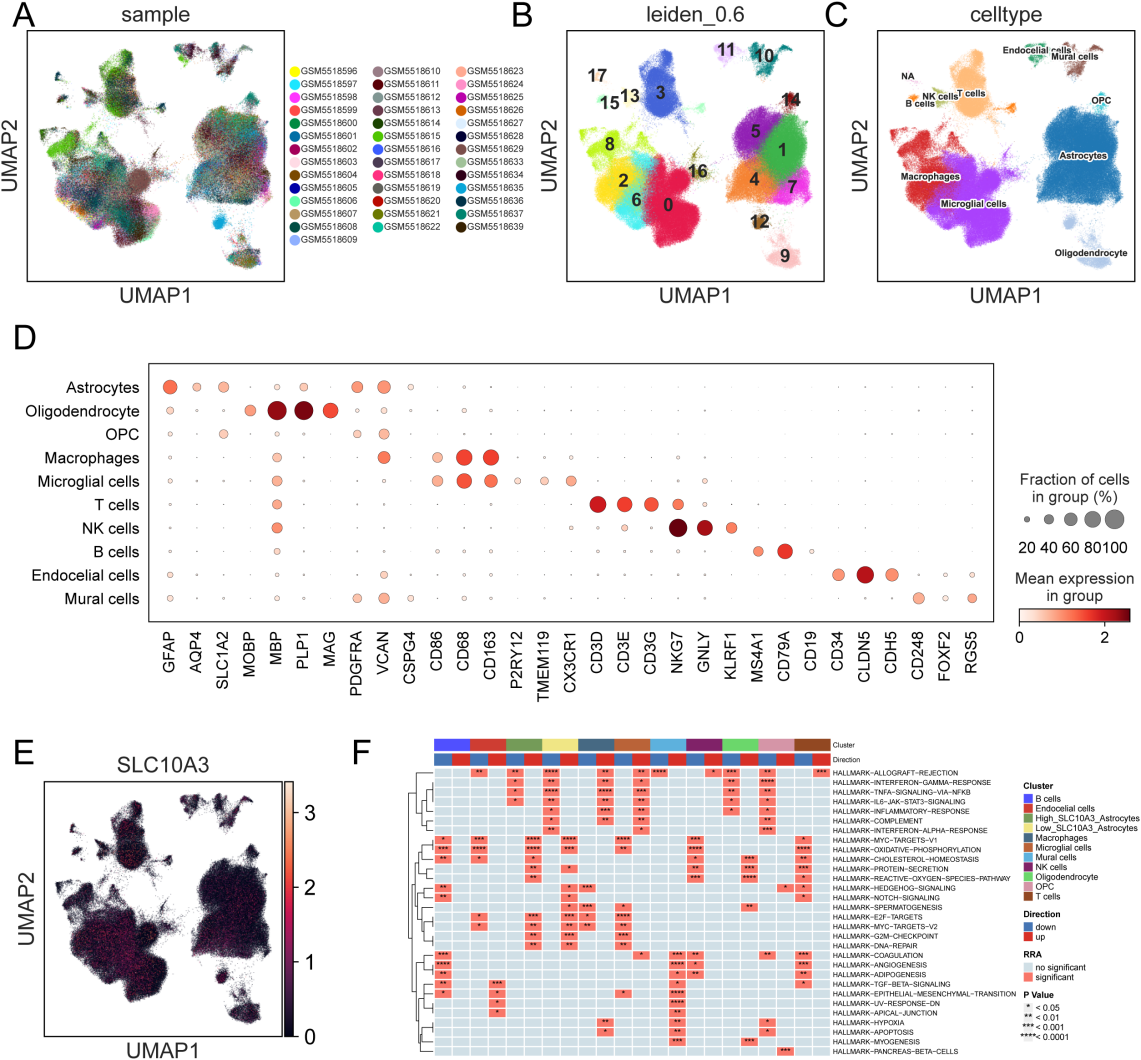
Molecular features of SLC10A3 at the single-cell level. **(A)** UMAP visualization of the integration of ten samples. **(B)** UMAP visualization of cell clustering. **(C)** UMAP visualization of clustering of ten cell types. **(D)** Identified marker genes in the ten cell types. **(E)** UMAP visualization of visualization of cells with SLC10A3 expression. **(F)** iGSEA analyzes the scores of different cell hallmark gene sets.

Using ‘Scanpy’ and ‘scVI’, we performed single-cell RNA sequencing analysis on 40 glioblastoma samples. All samples were perfectly integrated (**Figure 2A**) and the cells were clustered into 18 distinct populations (**Figure 2B**). These clusters were annotated based on cell marker genes, such as microglia, macrophages, T cells, natural killer (NK) cells, B cells, endothelial cells, oligodendrocytes, plasma cells, astrocytes, and oligodendrocyte precursor cells (OPCs) (**Figures 2C, D**). SLC10A3 expression was predominantly elevated in astrocytes and macrophages (**Figure 2E**). Tumor-associated astrocytes were further stratified into high- and low-expression subgroups based on the median expression level of SLC10A3. “irGSEA” analysis revealed that in SLC10A3-low astrocytes, the HALLMARK INFLAMMATORY RESPONSE, HALLMARK COMPLEMENT, and HALLMARK INTERFERON ALPHA RESPONSE signaling pathways were downregulated, whereas in SLC10A3-high cells, the HALLMARK REACTIVE OXYGEN SPECIES PATHWAY was upregulated (**Figure 2F**).

### 3.5 Cell-cell interactions associated with SLC10A3

**Figure 3:**
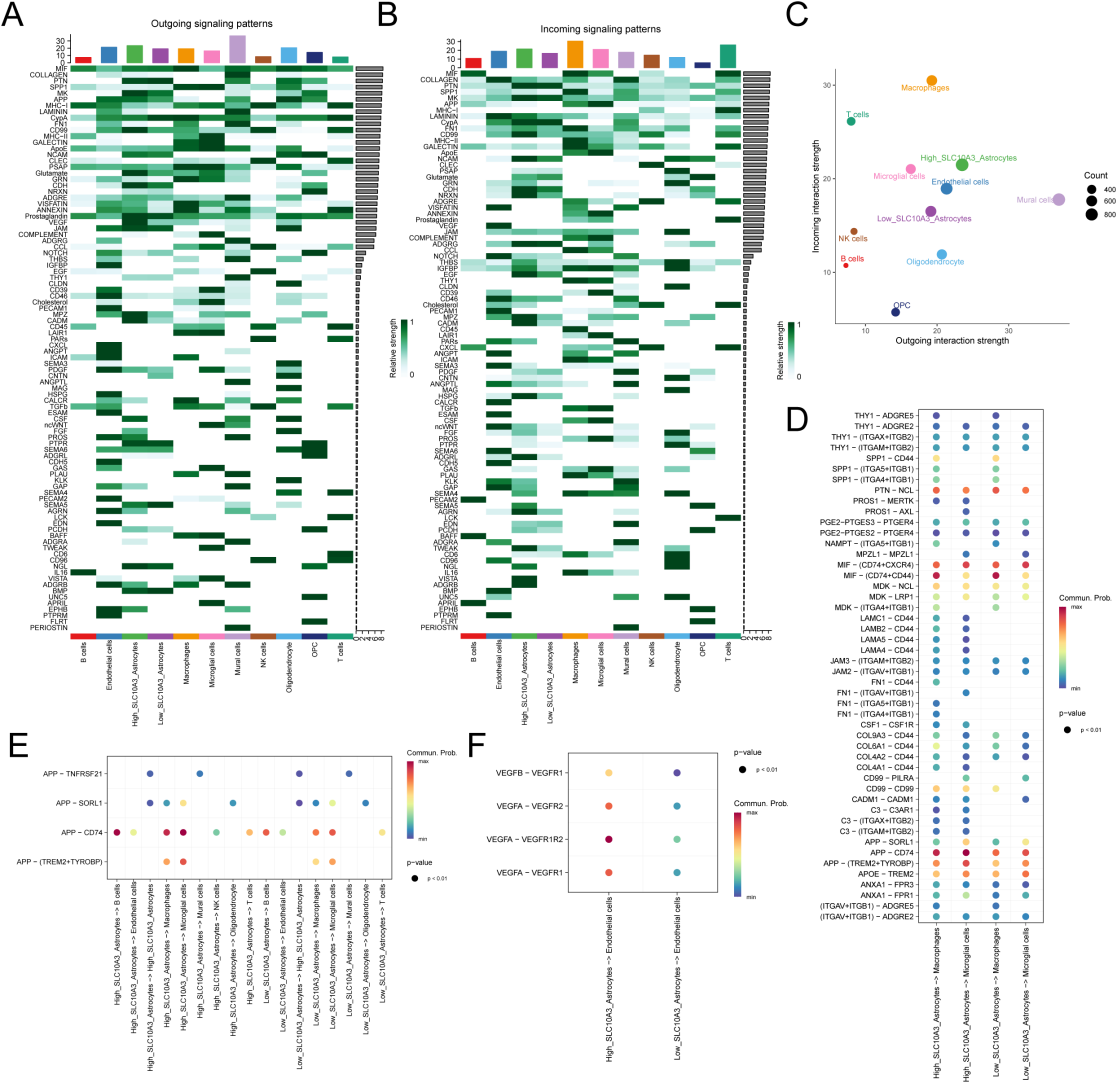
Cell-Cell Interactions Associated with SLC10A3. **(A)** Outgoing signaling patterns of different cells. **(B)** Incoming signaling patterns of different cells. **(C)** The signal strength of incoming and outgoing interactions in different cell types. **(D)** The cell signaling communication between high and low SLC10A3 astrocytes and macrophages/microglia. **(E)** Communication patterns of APP signaling between high and low SLC10A3 astrocytes and other cells. **(F)** Communication patterns of VEGFA signaling between high and low SLC10A3 astrocytes and endothelial cells.

**Figure 4:**
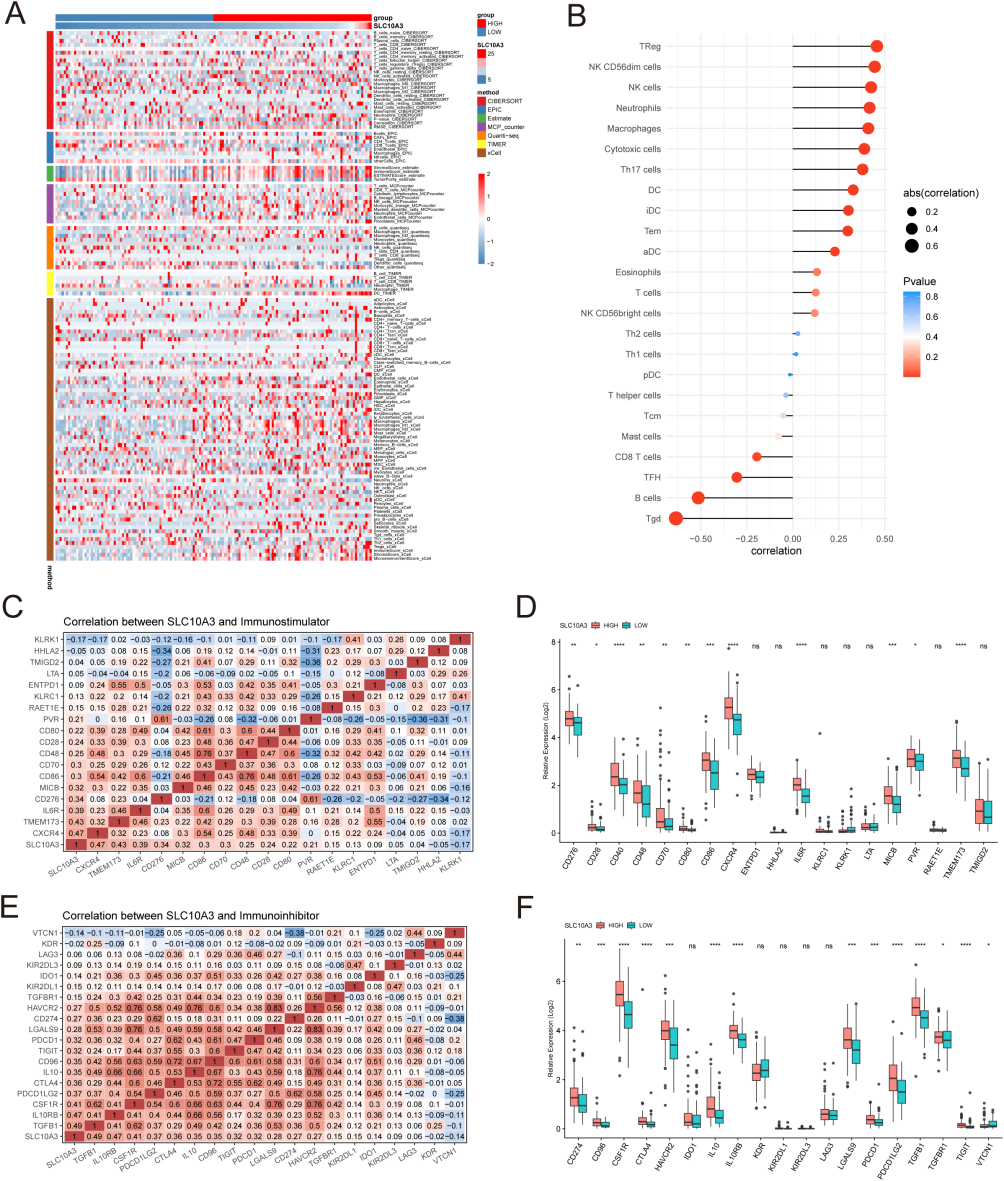
Immune infiltration analysis. **(A)** Heat map of immune cell infiltration in the high SLC10A3 group and low SLC10A3 group. **(B)** Correlation heat map between 24 immune cell infiltrates and SLC10A3 expression. **(C)** Correlation heat map between SLC10A3 and immune-promoting-related genes. **(D)** Expression of immune-promoting-related genes in the high SLC10A3 group and low SLC10A3 group. **(E)** Correlation heat map between SLC10A3 and immune-suppressive-related genes. **(F)** Expression of immune-suppressive-related genes in the high SLC10A3 group and low SLC10A3 group.(*p < 0.05, **p < 0.01, ***p < 0.001, ****p < 0.0001).

Cell Chat analysis of SLC10A3-high and SLC10A3-low astrocytes revealed significant differences in intercellular communications. In SLC10A3-high astrocytes, both outgoing (e.g., MK, APP, CDH, VEGF, MPZ, CADM) and incoming (e.g., NCAM, CD99, IGFBP, EGF, and ADGRL) signaling were markedly enhanced (**Figures 3A, B**). Furthermore, SLC10A3-high astrocytes exhibited greater communication network complexity and interaction strength than SLC10A3-low astrocytes (**Figure 4C**). Differential signaling pathways were identified in these two subsets. SLC10A3-high astrocytes showed stronger interactions with macrophages/microglia via the MIF, MDK, LAMC1, LAMB2, and FN1 signaling axes (**Figure 3D**). Additionally, enhanced APP signaling in T cells and VEGF signaling in endothelial cells were observed in SLC10A3-high astrocytes (**Figures 3E, F**).

### 3.6 Immune infiltration and mutational landscape of SLC10A3 in GBM

Through comprehensive analysis of the TCGA glioblastoma cohort using the IOBR package, we observed significantly elevated immune cell infiltration in SLC10A3-high patients compared to SLC10A3-low patients, as demonstrated by both heatmap visualization and ESTIMATE algorithm scores showing higher StromalScore, ImmuneScore and ESTIMATEScore in the high-expression group, with multiple computational methods consistently indicating increased macrophage infiltration (**Figure 4A**). ssGSEA analysis of 24 immune cell types revealed strong positive correlations between SLC10A3 expression and immunosuppressive populations including Tregs (r=0.490, P<0.001), NK CD56dim cells (r=0.472, P<0.001) and macrophages (r=0.450, P<0.001), and negative correlations with B cells and γδ T cells (**Figure 4B**). Further examination of immunomodulators demonstrated that SLC10A3 expression positively correlated with both immune stimulators (CXCR4, TMEM173, IL6R, CD276, MICB, CD86 and CD70) and inhibitors (TGFB1, IL10RB, CSF1R, PDCD1LG2, CTLA4, IL10 and CD96), and differential expression analysis confirmed significantly higher levels of key stimulators (CD276, CD40, CD48 and CXCR4) and inhibitors (CD274, CSF1R, CTLA4, PDCD1 and PDCD1LG2) in SLC10A3-high tumors (**Figures 4C–F**). Genomic characterization revealed distinct mutation patterns between the groups, with SLC10A3-high tumors exhibited more frequent alterations in TP53, EGFR and PTEN, along with specific enrichment of NF1 and STAG2 mutations, whereas SLC10A3-low tumors showed preferential mutations in PCDH11X and TRPV6 (**Supplementary Figures 3A–C**), suggesting an association between SLC10A3 expression and an immunosuppressive tumor microenvironment with distinct genomic features.

### 3.7 Upregulation of SLC10A3 expression in glioma tissues and cell lines

**Figure 5:**
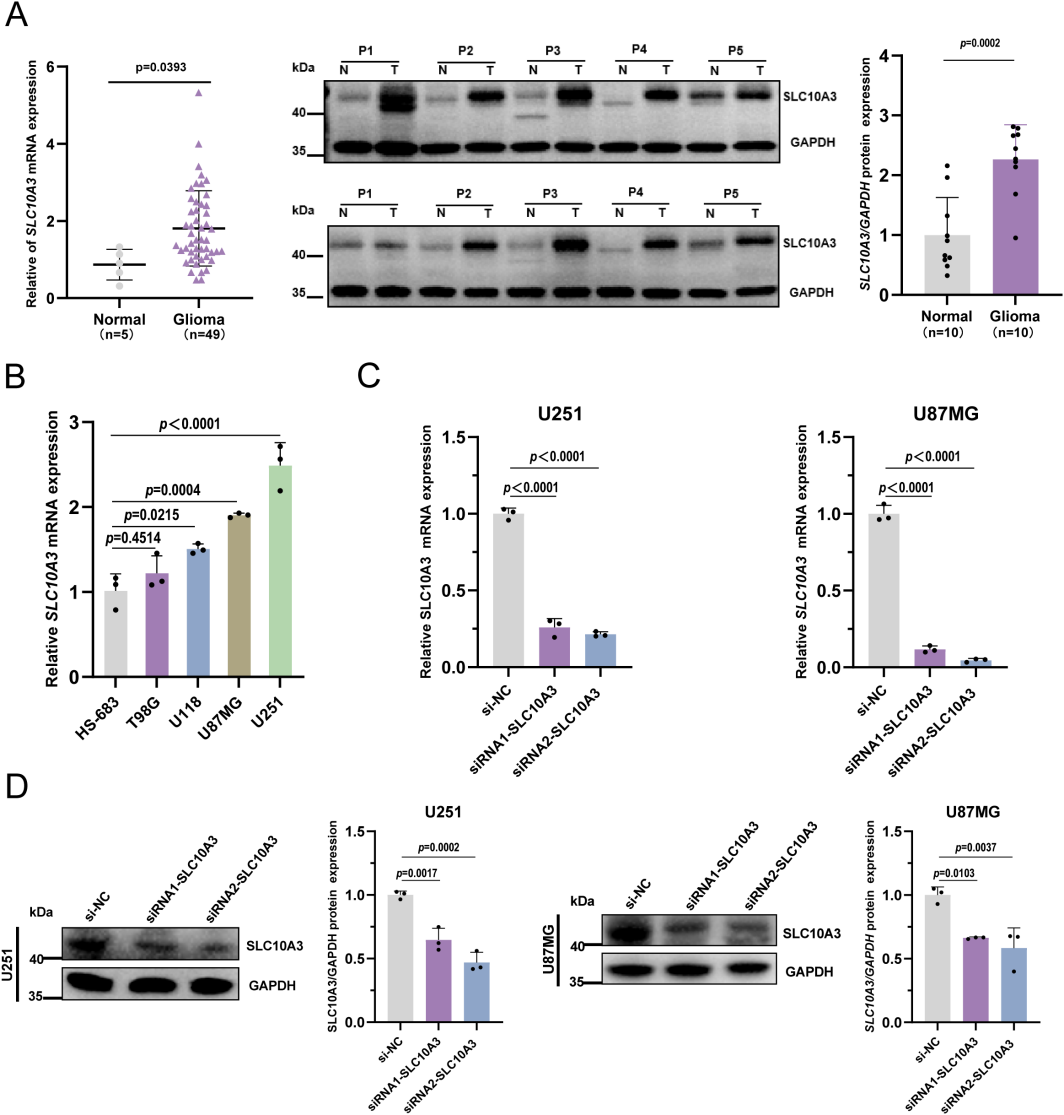
SLC10A3 is highly expressed in glioma. **(A)** Levels of SLC10A3 in glioma detected by qRT-PCR and Western blot. **(B)** Levels of SLC10A3 in different glioma cells detected by qRT-PCR. **(C, D)** After U251 and U87MG cells were transfected with siSLC10A3 or siNC for 48 h, the mRNA and protein expression of SLC10A3 was analyzed by qRT-PCR and Western blot assay.

To further validate the bioinformatics analysis results, we collected 49 glioma tissue samples and 5 non-tumor brain tissue samples (as normal controls) from Jiangxi Provincial Cancer Hospital. SLC10A3 mRNA levels were detected by RT-qPCR, which showed that SLC10A3 was significantly upregulated in glioma tissues compared with non-tumor controls. Additionally, Western blot analysis of 10 representative glioma tissues and their paired non-tumor tissues confirmed that SLC10A3 protein expression was consistent with the mRNA trend, aligning with the bioinformatics findings (**Figure 5A**).

Next, we measured SLC10A3 mRNA expression in different glioblastoma (GBM) cell lines. The results showed that SLC10A3 levels were significantly higher in U251 and U87MG cells than in other GBM cell lines (**Figure 5B**). Given their high SLC10A3 expression, U251 and U87MG cells were selected for subsequent experiments.

In these two cell lines, we transfected two SLC10A3-targeting siRNAs (si-SLC10A3–1 and si-SLC10A3-2) to specifically suppress SLC10A3 expression. RT-qPCR and Western blot confirmed efficient knockdown of SLC10A3 at both mRNA and protein levels (**Figures 5C, D**).

### 3.8 Inhibition of SLC10A3 activity or expression induces S phase cell cycle arrest and promotes glioma cell proliferation inhibition

**Figure 6:**
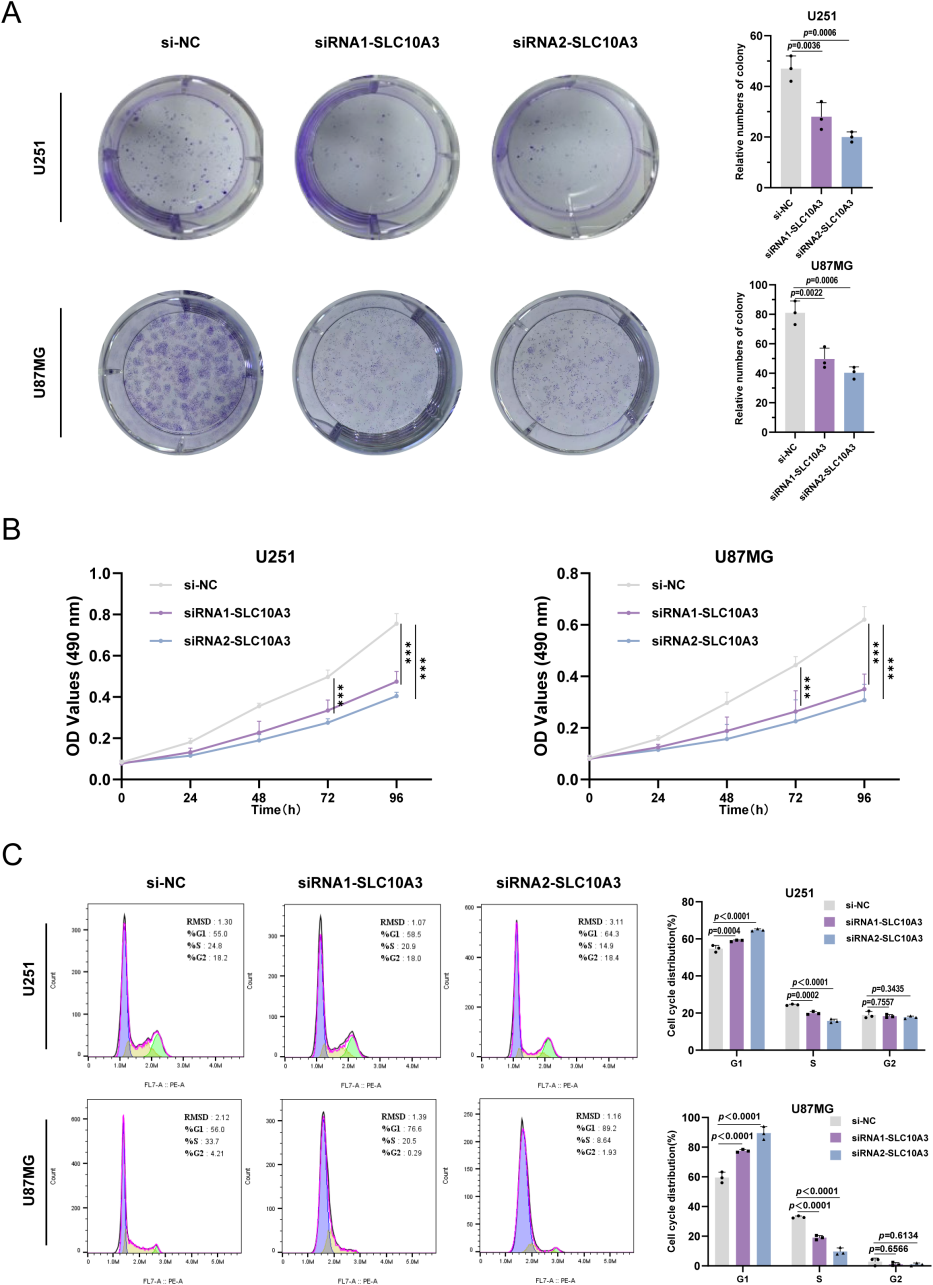
Inhibition of SLC10A3 activity or expression induces S-phase cell cycle arrest and promotes inhibition of glioma cell proliferation. **(A)** Colony formation was analyzed in U251 and U87MG cells. **(B)** MTS analysis was performed on U251 and U87MG cells. **(C)** Flow cytometry analysis of cell cycle distribution of control, U251 and U87MG cells treated with siNC or siSLC10A3 for 48 h. (*p < 0.05, **p < 0.01, ***p < 0.001, ****p < 0.0001).

In the colony formation assay, SLC10A3 knockdown resulted in a reduced number of colonies, indicating impaired clonogenic capacity of glioma cells (**Figure 6A**). Moreover, MTS assay revealed that si-SLC10A3 slowed the proliferation rate of glioma cells, further supporting a role for SLC10A3 in promoting cell growth (**Figure 6B**). Consistently, flow cytometric analysis demonstrated cell cycle arrest: compared to the control group, the proportion of cells in the G1 phase was increased while that in the S phase was decreased, suggesting inhibition of cell cycle progression (**Figure 6C**).

### 3.9 SLC10A3 affects the invasion and migration of glioma cells

**Figure 7:**
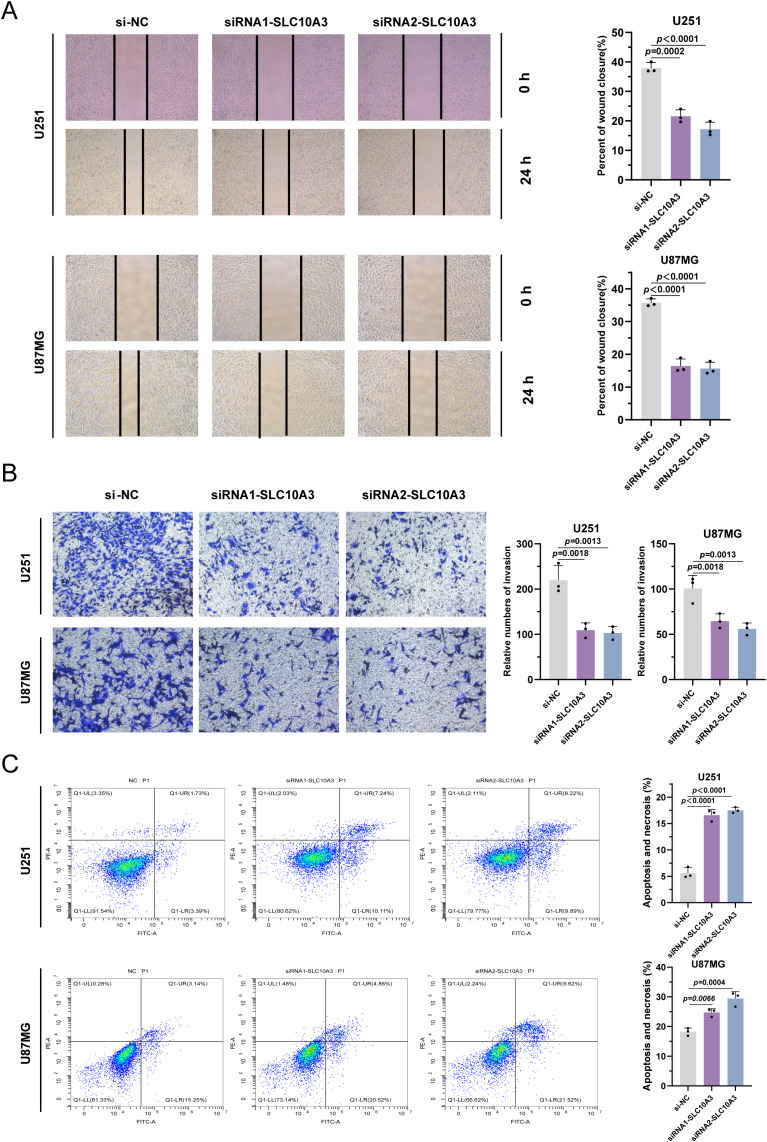
SLC10A3 affected the invasion and migration of glioma cells. **(A)** Wound healing was performed to determine the migration ability of U251 and U87MG cells. **(B)** Transwell assay was used to evaluated the migration of U251 and U87MG cells. **(C)** U251, U87MG cells were treated with siNC or siSLC10A3 for 48 h. A Flow cytometry assay showed the apoptosis of glioma. **P < 0.01, ***P < 0.001.

To investigate the effect of SLC10A3 on glioma cell migration and invasion, we performed wound healing and Transwell assays. In the wound healing assay, knockdown of SLC10A3 significantly reduced the migration rate of U251 and U87MG cells, indicating impaired migratory capacity (**Figure 7A**). Consistently, Transwell assay revealed a marked reduction in the invasive potential of SLC10A3-deficient cells, suggesting that SLC10A3 is required for glioma cell invasion (**Figure 7B**).

In addition to its effects on migration and invasion, apoptosis analysis by Annexin V-FITC/PI staining and flow cytometry showed that SLC10A3 silencing increased the proportion of apoptotic cells (**Figure 7C**). Collectively, these data suggest that SLC10A3 promotes glioma progression by enhancing cell migration, invasion, and survival.

### 3.10 The effect of SLC10A3 silencing on M2-like macrophages in glioma cell lines

**Figure 8:**
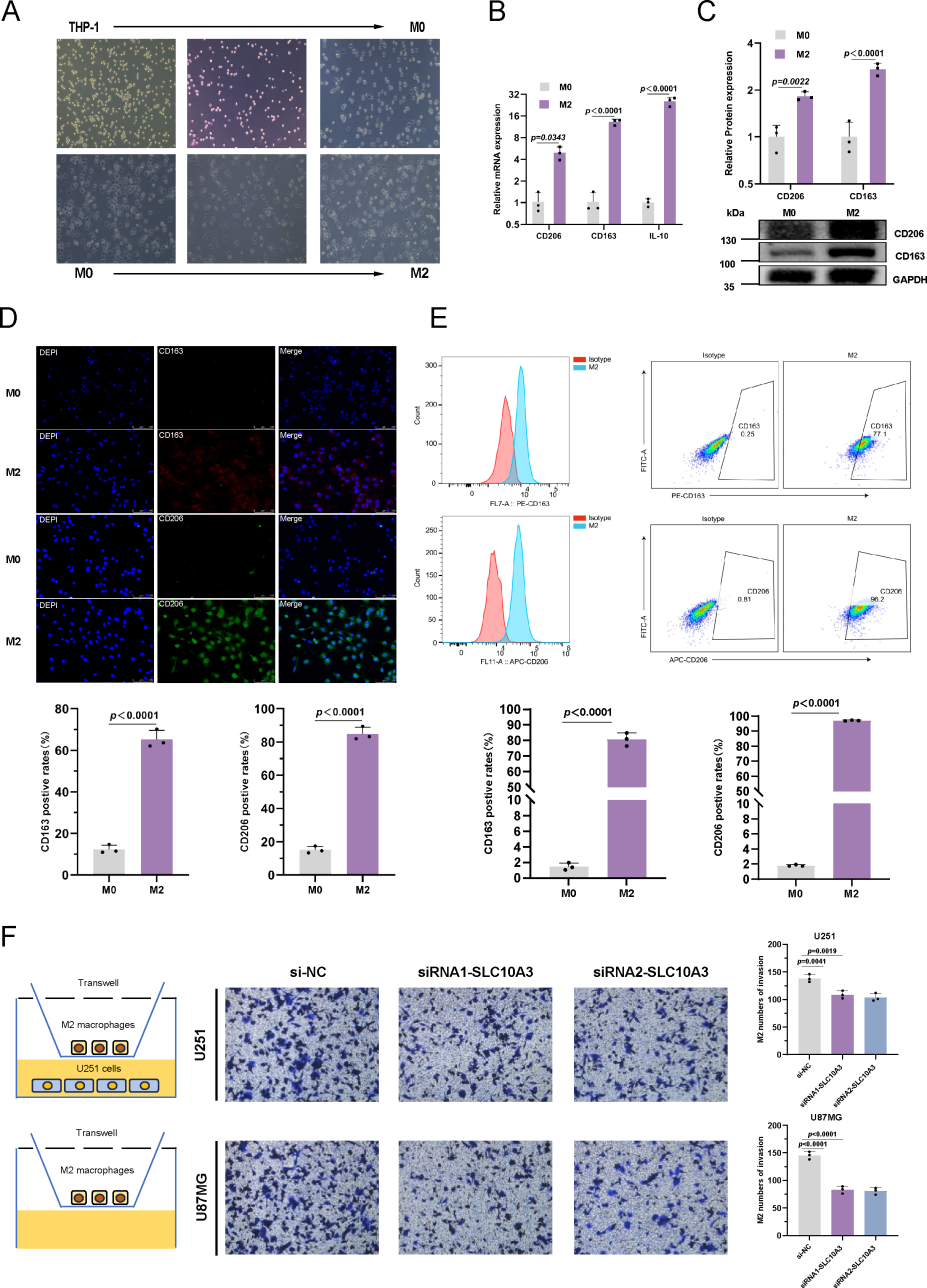
The effect of SLC10A3 silencing on M2 macrophages in glioma cell lines **(A)** Thp-1 polarized to the morphological changes of M2 macrophage. **(B)** Levels of CD206, CD163, IL-10 in M2 macrophage was analyzed by qRT-PCR. **(C)** Levels of CD206, CD163 in M2 macrophage were detected by qRT-PCR and Western blot. **(D)** Immunofluorescence assay of glioma cells were transfected with siNC or siSLC10A3. The proportion of M2 macrophages (CD163+/CD206+) was quantified by flow cytometry. Isotype controls were PE-mouse IgG1, κ(clone MOPC-21) for CD163 and APC-mouse IgG1, κ(clone MOPC-21) for CD206, each used at the same dilution as the corresponding antibody (1:100). **(F)** Macrophages THP-1 were co-cultured with glioma cells U251 and U87MG, and Transwell migration assay was used to detect the effect of SLC10A3-deficient U251 and U87MG cells on the migration ability of THP-1 cells.

To investigate whether SLC10A3 in glioma cells regulates M2 macrophages, we first established a THP-1-derived M2-like macrophages model and validated its polarization status (a prerequisite for subsequent experiments). To this end, THP-1 cells were polarized into M2-like macrophages (**Figure 8A**), and their identity was confirmed using M2-specific markers CD163 and CD206. Compared with M0 unpolarized macrophages, RT-qPCR and Western blot showed significantly increased CD163 and CD206 levels at both mRNA and protein levels in M2-like macrophages; immunofluorescence further confirmed that CD163 and CD206 were primarily localized in M2-like macrophages (**Figures 8B–D**). Consistently, flow cytometric analysis revealed higher expression of CD163 and CD206 in M2 macrophages than in M0 cells, confirming successful polarization (**Figure 8E**).

Subsequently, we explored whether SLC10A3 in glioma cells affects M2 macrophage migration. In a Transwell co-culture system, M2-polarized THP-1 cells were seeded in the upper chamber, and SLC10A3-silenced U251/U87MG cells (si-SLC10A3–1 and si-SLC10A3-2) or control cells (si-NC) were seeded in the lower chamber. After 48 hours of co-culture, M2-like macrophages migration in the si-SLC10A3 groups was significantly reduced compared with the si-NC group (**Figure 8F**).

These results suggest that SLC10A3 overexpression in glioma cells may promote M2 macrophage migration into the glioma tumor microenvironment (TME), thereby contributing to the immunosuppressive niche.

### 3.11 The effect of SLC10A3 knockdown on the tumorigenic ability of glioma cells

**Figure 9:**
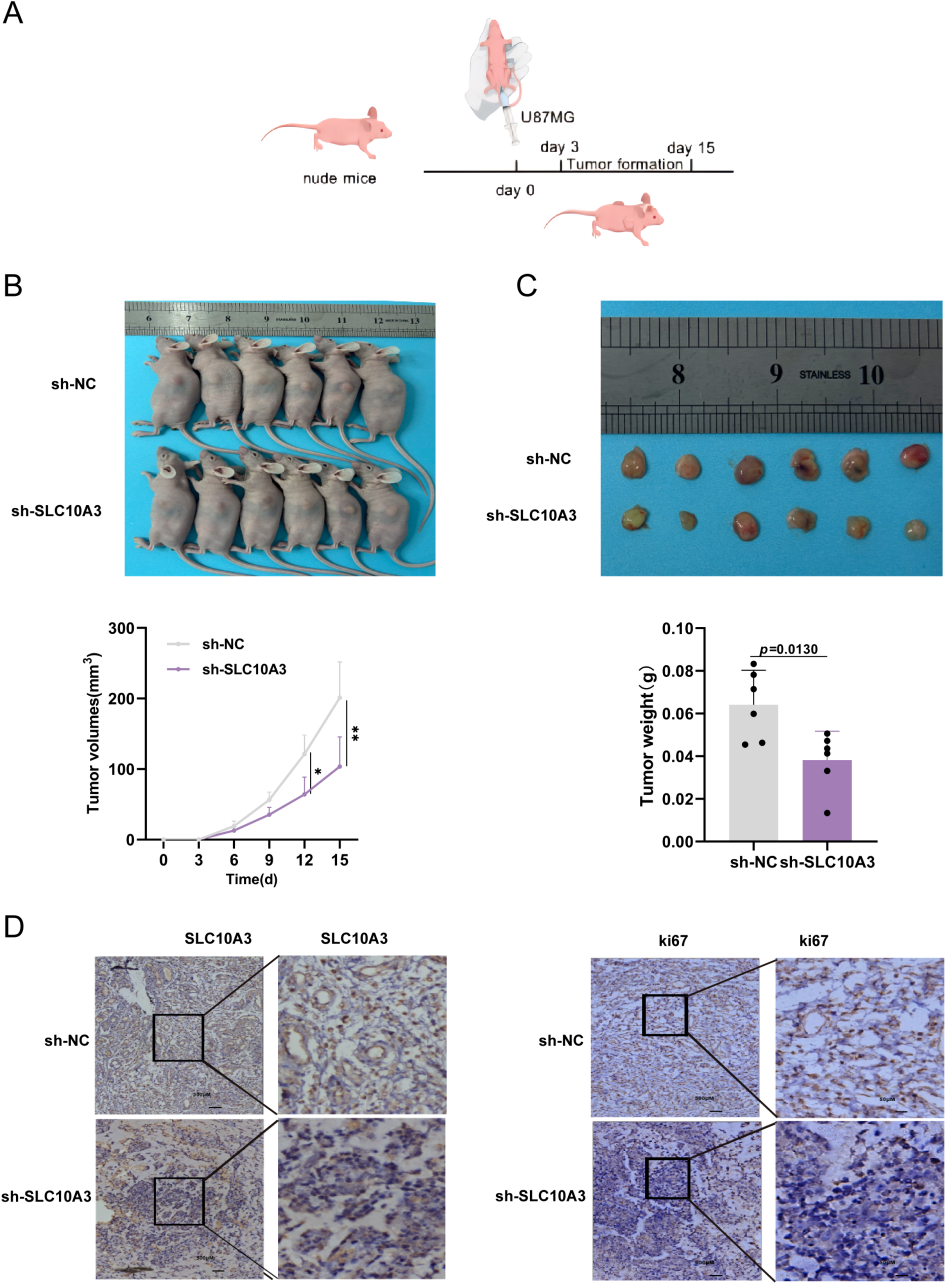
**(A)** Nude mouse tumorigenesis assay to verify the *in vivo* effect of SLC10A3. **(B, C)** Comparison of tumor volume and tumor weight of 5 pairs of nude mice treated with NC and sh-SLC10A3. **(D)** Immunohistochemistry was performed to evaluate the expression of SLC10A3 and Ki67 in tumor samples. Experimental data are expressed as mean ± SEM (n = 6).

To investigate the effect of SLC10A3 on the *in vivo* tumorigenic ability of glioma cells, stable U87MG cells from the negative control (NC) group and SLC10A3 short hairpin RNA (sh-SLC10A3) group were subcutaneously injected into 4–6-week-old nude mice (n = 6 per group; **Figure 9A**).

The results showed that SLC10A3 knockdown significantly reduced tumor volume and slowed tumor growth compared with the NC group—key features of *in vivo* tumorigenic potential—indicating impaired tumor-forming capacity *in vivo* (**Figure 9B**).

Collectively, Immunohistochemical (IHC) staining further revealed a significant decrease in the proportion of Ki67-positive tumor cells in the sh-SLC10A3 group, which directly reflects reduced proliferative activity and provides additional evidence for the impaired tumorigenic ability caused by SLC10A3 knockdown (**Figure 9C**).

## 4 Discussion

Glioblastoma (GBM) is an intracranial tumor with the highest degree of malignancy. Owning to its high recurrence rate and widespread temozolomide resistance, the prognosis of patients is relatively poor (34). Although immunotherapies such as immune checkpoint inhibitors (ICIs) have achieved good therapeutic effects in various cancers, such as melanoma and lung cancer, in a phase III clinical trial of nivolumab for treating GBM, monotherapy with PD-1 did not improve the prognosis of patients (35). The main reasons for the failure of immunotherapy are the immunosuppressive microenvironment of GBM and the reduction in immune cells caused by radiotherapy and chemotherapy (36, 37). Therefore, identifying specific molecular markers related to the progression of GBM, especially genes that affect the function of immune cells, is of great significance for improving the current situation of GBM immunotherapy.

Our study found that SLC10A3 was highly expressed in GBM, low-grade glioma (LGG), adrenocortical carcinoma (ACC), breast invasive carcinoma (BRCA), cervical squamous cell carcinoma (CESC), cholangiocarcinoma (CHOL), colon adenocarcinoma (COAD), diffuse large B-cell lymphoma (DLBC), esophageal carcinoma (ESCA), head and neck squamous cell carcinoma (HNSC), kidney renal clear cell carcinoma (KIRC), liver hepatocellular carcinoma (LIHC), lung squamous cell carcinoma (LUSC), pancreatic adenocarcinoma (PAAD), rectum adenocarcinoma (READ), stomach adenocarcinoma (STAD), testicular germ cell tumors (TGCT), and thymoma (THYM) tumors. Subsequently, we further confirmed the significantly high expression of SLC10A3 in GBM samples through GBM transcriptome data from TCGA and CGGA, reverse transcription quantitative polymerase chain reaction (RT-qPCR), and Western blotting. Survival analysis using TCGA and CGGA datasets revealed that patients with high SLC10A3 expression had a poor prognosis. Based on these findings, SLC10A3 may be involved in the malignant progression of GBM.

Next, we analyzed the signaling pathways related to SLC10A3 in GBM to understand its possible carcinogenic mechanisms. Differential analysis was performed according to the median value of SLC10A3 expression and gene set enrichment analysis (GSEA) of the differentially expressed genes showed that the signaling pathways of HALLMARK E2F TARGETS, HALLMARK G2M CHECKPOINT, HALLMARK MYC TARGETS V1 and HALLMARK OXIDATIVE PHOSPHORYLATION were upregulated, whereas the pathways of HALLMARK ALLOGRAFT REJECTION, HALLMARK EPITHELIAL MESENCHYMAL TRANSITION, HALLMARK INFLAMMATORY RESPONSE, HALLMARK INTERFERON GAMMA RESPONSE and HALLMARK TNFA SIGNALING VIA NFKB were downregulated. In addition, through Gene Ontology (GO) and Kyoto Encyclopedia of Genes and Genomes (KEGG) enrichment analysis, we found that SLC10A3 was related to biological functions and signaling pathways such as humoral immune response, glial cell differentiation and development, integrin binding, PI3K−Akt signaling pathway, TNF signaling pathway, NF−κB signaling pathway, and HIF−1 signaling pathway. Based on these results, SLC10A3 may play a key regulatory role in the proliferation, invasion and migration of glioblastoma cells. To clarify the correlation between SLC10A3 and the proliferation, invasion and migration abilities of glioma cells, we conducted functional verification using MTS, EDU, and transwell assays. The results showed that after knocking out SLC10A3, the DNA synthesis ability of U251 and T98G cells was significantly weakened, and the cell proliferation activity was inhibited. Further functional analysis showed that deletion of SLC10A3 significantly reduced the invasion and migration abilities of the two glioma cell lines. Mechanistic studies found that inhibiting the expression or activity of SLC10A3 could induce cell cycle arrest in the S phase, thereby inhibiting the proliferation of glioma cells. At the same time, this inhibitory effect could significantly increase the apoptosis rate of glioma cells. In addition, *in vivo* experiments showed that inhibiting the expression of SLC10A3 significantly inhibited the growth of subcutaneous tumors, with a significant reduction in tumor volume and weight. These results indicate that SLC10A3 plays a crucial role in the proliferation, invasion and migration of glioblastoma, and its inhibition may become a new strategy for treating glioblastoma.

Given that SLC10A3 was found to be related to the immune microenvironment in GBM in the bulk transcriptome, we further analyzed SLC10A3 in the single-cell dataset. The results showed that SLC10A3 was widely expressed in astrocytes, macrophages and microglia. Subsequently, we divided astrocytes into high- and low SLC10A3-expressing astrocytes according to the expression level of SLC10A3, and analyzed the differences between the two cell types. We found that the signaling pathways of HALLMARK INFLAMMATORY RESPONSE, HALLMARK COMPLEMENT and HALLMARK INTERFERON ALPHA RESPONSE were downregulated in cells with low SLC10A3 expression. These results indicate that the expression of SLC10A3 is related to the immune microenvironment of glioma. Further cell communication analysis revealed differences in communication between astrocytes with high and low SLC10A3 expression, macrophages, and microglia. High SLC10A3 expression of astrocytes enhances their interactions with macrophages/microglia through the MIF, MDK, LAMC1, LAMB2 and FN1 pathways. Studies have shown that MIF signaling in the tumor microenvironment (TME) contributes to the anti-inflammatory, immune evasion and immune tolerance phenotypes of innate and adaptive immune cells (38). MDK is a secreted protein that is involved in the transformation of macrophages into a tolerant phenotype in melanoma and CRC, leading to the dysfunction of CD8+ T cells, thus promoting the formation of an immunosuppressive microenvironment (39, 40). These results indicated that SLC10A3 is related to the immune microenvironment of GBM cells. In addition, we found in the transcriptome data analysis that the Stromal, Immune and ESTIMATE Scores of the group with high SLC10A3 expression were all higher than those of the group with low SLC10A3 expression, and multiple immune infiltration analysis algorithms showed that the infiltration of macrophages in the group with high SLC10A3 expression was higher. SLC10A3 was positively correlated with regulatory T cells and macrophages. By analyzing the correlation between the expression of SLC10A3 and cell marker genes of immune cells, we found that SLC10A3 had different regulatory effects on the functions of different immune infiltrating cells. SLC10A3 positively correlated with the immunosuppressive genes TGFB1, IL10RB, CSF1R, PDCD1LG2, CTLA4 and IL10. In addition, our results showed that through the immune co-culture experiment, in the absence of SLC10A3, the migration ability of M2 macrophages was inhibited, suggesting a correlation between the overexpression of SLC10A3 in glioma and the immunosuppressive microenvironment. This indicates that combining the inhibition of SLC10A3 with immunotherapy may have potential therapeutic value and provide a potential approach to improve the effectiveness of cancer immunotherapy.

Although our co-culture experiments demonstrated that SLC10A3 promotes M2 macrophage migration, the precise molecular mediators remain unclear. Based on our single-cell communication analysis, signaling axes including MIF, MDK, and ECM-associated ligands may represent potential downstream mechanisms. Future studies involving cytokine profiling, neutralizing antibodies, or receptor blockade experiments will be required to validate these pathways. Furthermore, we acknowledge that *in vitro* co-culture systems cannot fully recapitulate the spatial and cellular complexity of the GBM tumor microenvironment *in vivo*.

As a heterologous membrane surface protein of the SLC family, SLC10A3 can be directly recognized and bound by monoclonal antibodies (mAbs), antibody-drug conjugates (ADCs), bispecific antibodies, etc., and can exert its functions without entering cells, making it an ideal antibody drug target. Existing studies have shown that SLC10A3 can inhibit ferroptosis through the STAT3/GPX4 pathway and drive the progression of GBM (41). In addition, in a study on liver cancer, SLC10A3 was associated with the expression of PD1 and PD-L1 (42). Therefore, based on previous studies and the results of this study, antibody drugs targeting SLC10A3 can effectively inhibit the growth, invasion, and immune escape of GBM and other malignant tumors through the dual mechanisms of inducing ferroptosis and remodeling the tumor immune microenvironment, which is a new anti-tumor therapeutic strategy with great translational value.

In summary, we identified SLC10A3 as a key driver of glioma progression, with its knockout suppressing malignant phenotypes and altering M2 macrophage infiltration. However, two critical limitations remain: the specific signaling molecules (cytokines/chemokines) governing this immune modulation are unknown, and established cell lines may not fully represent primary GBM heterogeneity. To address these gaps, future research should extend validation to diverse GBM cell lines (e.g., LN229, A172, U373) and PDX models. This will not only establish the universality of SLC10A3’s function but also enable a deeper mechanistic dissection of how SLC10A3 fosters an immunosuppressive tumor microenvironment.

## Data Availability

The original contributions presented in the study are included in the article/**Supplementary Material**. Further inquiries can be directed to the corresponding authors.

## Supplementary material

Supplementary Figure 1 **(A)** Expression levels of SLC10A3 in different tumor types. **(B)** Hazard ratios of SLC10A3 expression in all TCGA cancers Cohort. B: Survival analysis of SLC10A3 in six types of cancers, including ACC, PAAD, KIRC, LGG, LAML, and COAD. **(C)** Kaplan-Meier survival curves for overall survival in TCGA ACC, PAAD, KIRC, LGG, LAML, and COAD cohorts stratified by SLC10A3 expression.

Supplementary Figure 2 **(A)** SLC10A3 expression is positively correlated with that of epithelial-mesenchymal transition (EMT) and extracellular matrix (ECM) remodeling-related genes. **(B)** SLC10A3 is enriched in hyperplastic blood vessels and microvascular proliferation regions of GBM (Ivy-GAP dataset).

Supplementary Figure 3 **(A, B)** Mutation profiles of the SLC10A3 low-expression group and high-expression group in TCGA-GBM data. **(C)** Differences in gene mutations between the SLC10A3 low-expression group and high-expression group (*P < 0.05, **P < 0.01).
