# Supplementary material for: SLC10A3 drives glioblastoma progression by remodeling the immunosuppressive microenvironment and promoting M2 macrophage migration
Source: Front Oncol. 2026 May 13;16:1742452. doi: 10.3389/fonc.2026.1742452 (PMC13212135; doi:10.3389/fonc.2026.1742452)
Supplement: Supplementary Figure 1 — (A) Expression levels of SLC10A3 in different tumor types. (B) Hazard ratios of SLC10A3 expression in all TCGA cancers Cohort. B: Survival analysis of SLC10A3 in six types of cancers, including ACC, PAAD, KIRC, LGG, LAML, and COAD. (C) Kaplan-Meier survival curves for overall survival in TCGA ACC, PAAD, KIRC, LGG, LAML, and COAD cohorts stratified by SLC10A3 expression. [file DataSheet1.docx]

Supplement figure


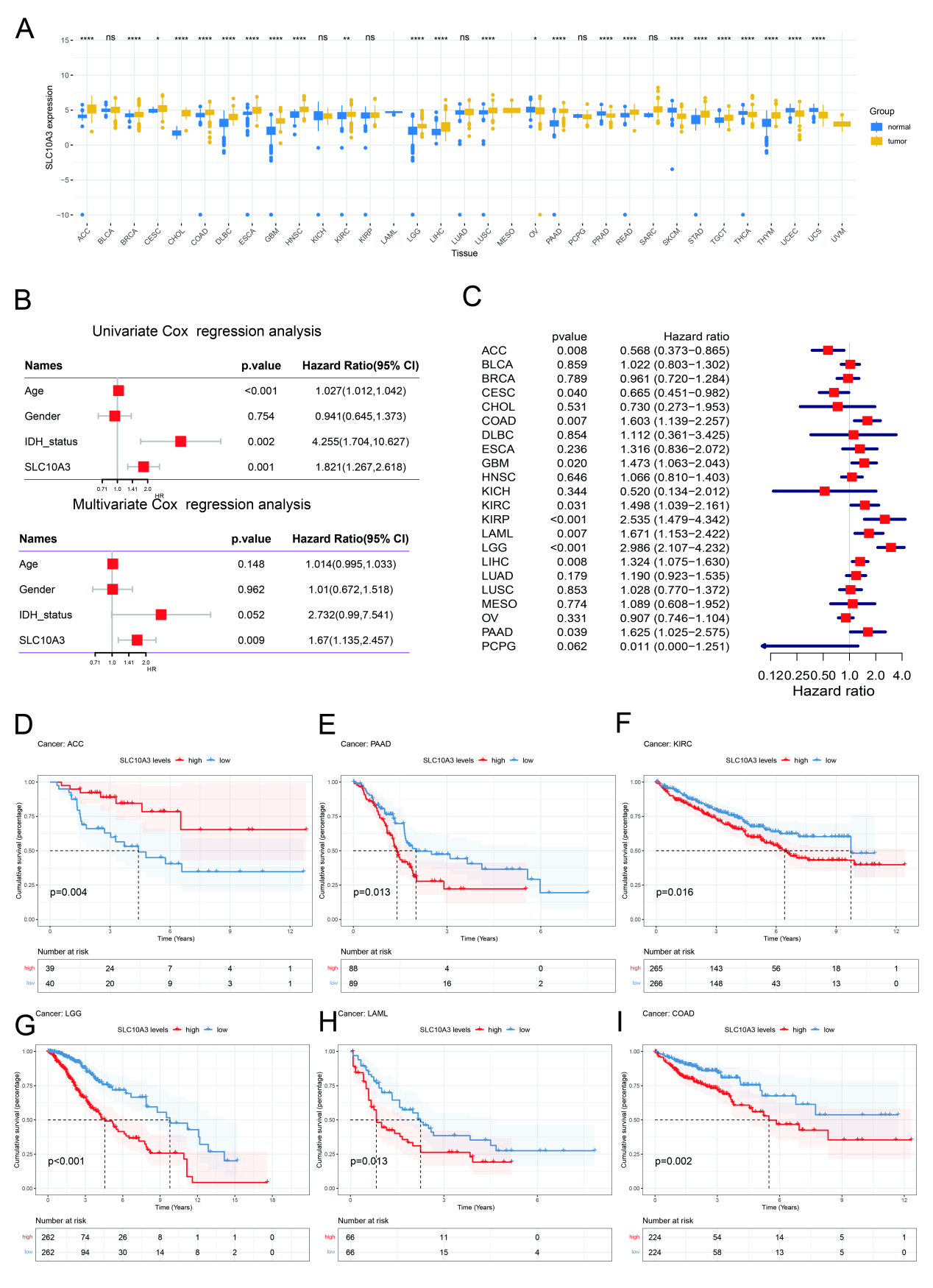


Supplement figure 1：A:Expression of SLC10A3 in pan-cancer. B:Univariate and multivariate regression analyses of age, gender, IDH status, and SLC10A3 in the TCGA cohort. C: Hazard ratios of SLC10A3 expression in all TCGA cancers Cohort. D: Survival analysis of SLC10A3 in six types of cancers, including ACC, PAAD, KIRC, LGG, LAML, and COAD.


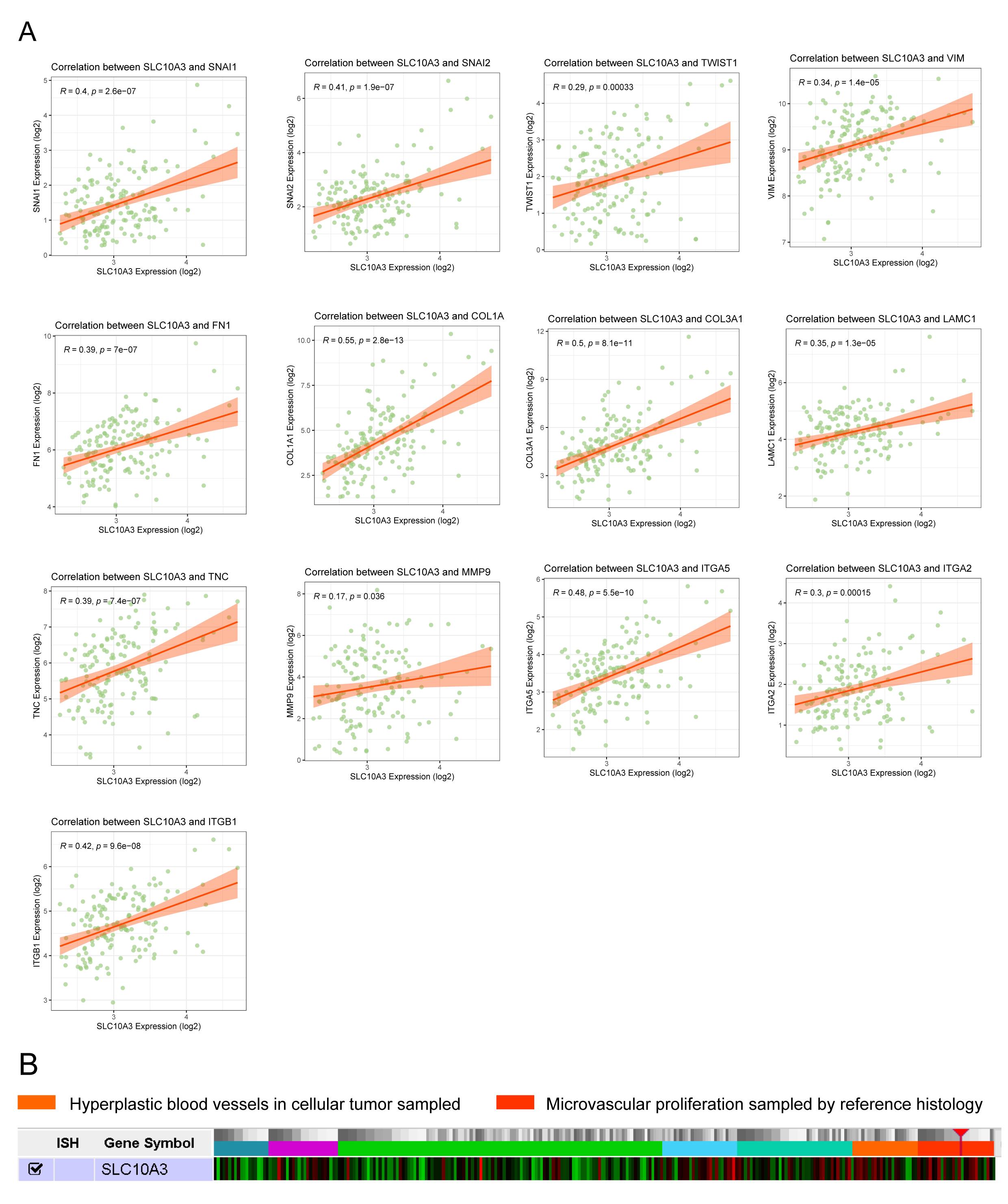


Supplement figure 2:(A) Correlation analysis between SLC10A3 and representative genes involved in epithelial-mesenchymal transition (EMT) and extracellular matrix (ECM) remodeling, including SNAI1, SNAI2, TWIST1, VIM, FN1, COL1A1, COL3A1, LAMC1, TNC, MMP9, ITGA5, ITGA2, and ITGB1. SLC10A3 exhibited a significant positive correlation with these genes, suggesting enhanced mesenchymal transition and ECM remodeling.(B) Regional expression distribution of SLC10A3 in glioblastoma analyzed using the Ivy Glioblastoma Atlas Project (Ivy-GAP) dataset. SLC10A3 was enriched in hyperplastic blood vessels and microvascular proliferation regions, in line with the characteristics of ECM remodeling.


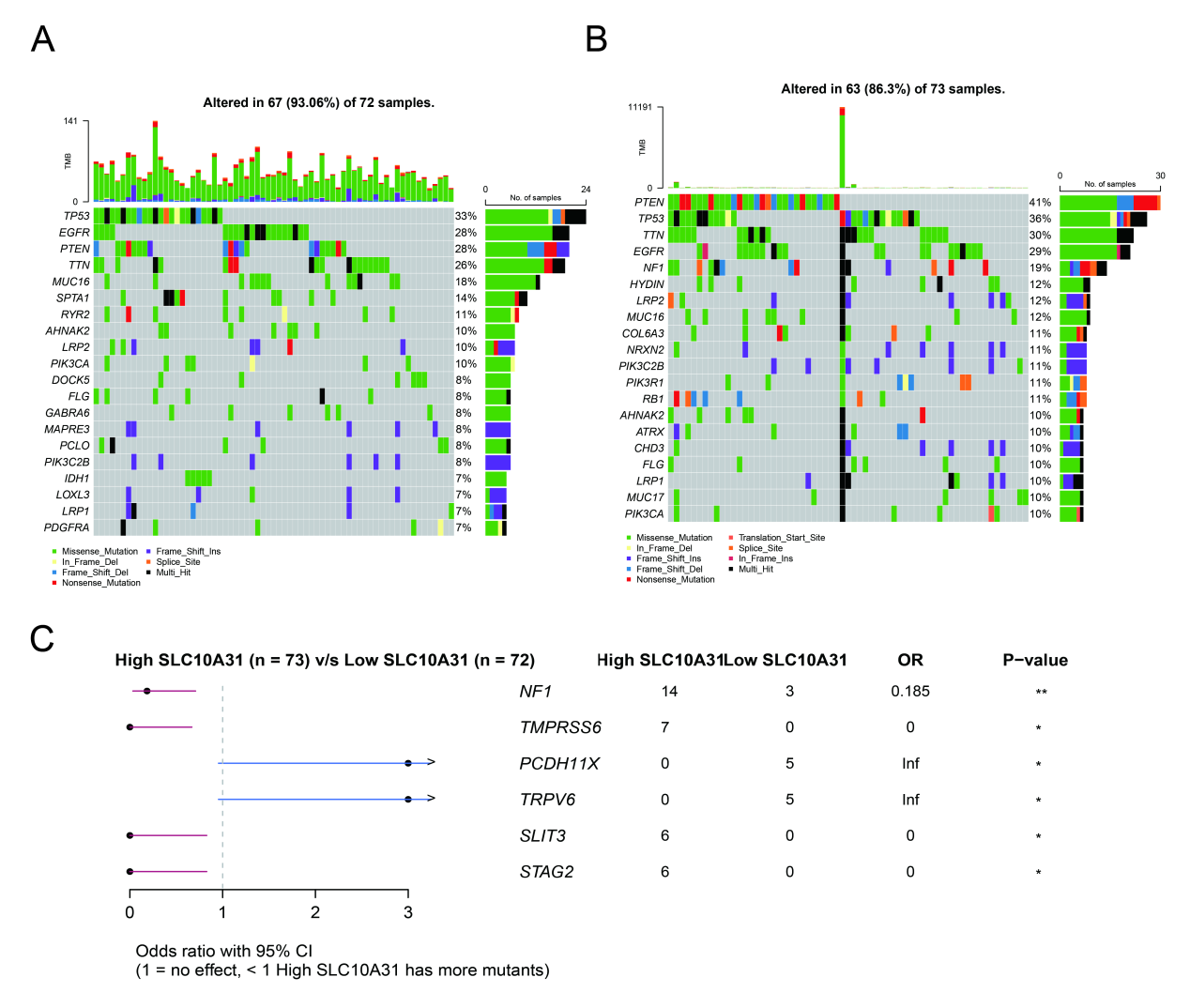


Supplement figure 3：A, B: Mutation profiles of the SLC10A3 low-expression group and high-expression group in TCGA-GBM data. C: Differences in gene mutations between the SLC10A3 low-expression group and high-expression group (*P < 0.05, **P < 0.01).
